# Lactate does not activate NF-κB in oxidative tumor cells

**DOI:** 10.3389/fphar.2015.00228

**Published:** 2015-10-13

**Authors:** Vincent F. Van Hée, Jhudit Pérez-Escuredo, Andrea Cacace, Tamara Copetti, Pierre Sonveaux

**Affiliations:** Pole of Pharmacology, Institut de Recherche Expérimentale et Clinique, Université Catholique de Louvain (UCL) Medical SchoolBrussels, Belgium

**Keywords:** cancer metabolism, mitochondria, lactate signaling, oxidative phosphorylation (OXPHOS), nuclear factor-κB, malate-aspartate shuttle, NADH, NAD(P)H oxidases (Nox)

## Abstract

The lactate anion is currently emerging as an oncometabolite. Lactate, produced and exported by glycolytic and glutaminolytic cells in tumors, can be recycled as an oxidative fuel by oxidative tumors cells. Independently of hypoxia, it can also activate transcription factor hypoxia-inducible factor-1 (HIF-1) in tumor and endothelial cells, promoting angiogenesis. These protumoral activities of lactate depend on lactate uptake, a process primarily facilitated by the inward, passive lactate-proton symporter monocarboxylate transporter 1 (MCT1); the conversion of lactate and NAD^+^ to pyruvate, NADH and H^+^ by lactate dehydrogenase-1 (LDH-1); and a competition between pyruvate and α-ketoglutarate that inhibits prolylhydroxylases (PHDs). Endothelial cells do not primarily use lactate as an oxidative fuel but, rather, as a signaling agent. In addition to HIF-1, lactate can indeed activate transcription factor nuclear factor-κB (NF-κB) in these cells, through a mechanism not only depending on PHD inhibition but also on NADH alimenting NAD(P)H oxidases to generate reactive oxygen species (ROS). While NF-κB activity in endothelial cells promotes angiogenesis, NF-κB activation in tumor cells is known to stimulate tumor progression by conferring resistance to apoptosis, stemness, pro-angiogenic and metastatic capabilities. In this study, we therefore tested whether exogenous lactate could activate NF-κB in oxidative tumor cells equipped for lactate signaling. We report that, precisely because they are oxidative, HeLa and SiHa human tumor cells do not activate NF-κB in response to lactate. Indeed, while lactate-derived pyruvate is well-known to inhibit PHDs in these cells, we found that NADH aliments oxidative phosphorylation (OXPHOS) in mitochondria rather than NAD(P)H oxidases in the cytosol. These data were confirmed using oxidative human Cal27 and MCF7 tumor cells. This new information positions the malate-aspartate shuttle as a key player in the oxidative metabolism of lactate: similar to glycolysis that aliments OXPHOS with pyruvate produced by pyruvate kinase and NADH produced by glyceraldehyde-3-phosphate dehydrogenase (GAPDH), oxidative lactate metabolism aliments OXPHOS in oxidative tumor cells with pyruvate and NADH produced by LDH1.

## Introduction

The lactate anion was recently identified as a tumor growth-promoting factor. In solid tumors, lactate is produced by different cell types that metabolically rely on anaerobic glycolysis (Kennedy and Dewhirst, 2010), aerobic glycolysis (Whitaker-Menezes et al., 2011; Dhup et al., 2012) or glutaminolysis (Vander Heiden et al., 2009) for survival and proliferation. Once produced, lactate is exported together with protons by passive monocarboxylate transporters (MCTs), among which MCT4 is particularly well adapted for lactic acid release (Dimmer et al., 2000; Ullah et al., 2006). If proton release and extracellular acidification have been well characterized to promote tumor progression, several studies also correlated high levels of lactate in clinical tumor specimen to tumor aggressiveness and progression to the metastatic stage (Walenta and Mueller-Klieser, 2004). One possible interpretation is that lactate levels merely reflect glycolytic activities in tumors, owing to the fact that a glycolytic metabolism offers several oncogenic advantages to tumor cells (Porporato et al., 2011). However, it is also now clearly established that the lactate anion itself directly promotes tumor progression. Lactate can indeed serve as an oxidative fuel, thereby supporting glycolytic-oxidative tumor cell cooperation (Sonveaux et al., 2008; Mendoza-Juez et al., 2012; Guillaumond et al., 2013; Kennedy et al., 2013) and fibroblasts-tumor cells metabolic relationships (Whitaker-Menezes et al., 2011). It can also act as a paracrine factor that activates lactate-sensitive signaling pathways. Collectively, these activities of lactate primarily depend on the expression and activity of the inward lactate transporter MCT1 at the surface of responsive cells that take up lactate.

In endothelial cells, lactate acts as a signaling agent that activates nuclear factor-κB (NF-κB) independently of hypoxia (Vegran et al., 2011, 2012). This signaling pathway of lactate involves MCT1-facilitated lactate uptake, lactate oxidation to pyruvate (the LDH-1/LDH-B reaction), and pyruvate-mediated inhibition of prolylhydroxylases (PHDs). It initiates the serial activation of inhibitor of NF-κB kinase β (IKK2/IKKβ), phosphorylation and degradation of inhibitor of NF-κB α (IκBα), and induction of the transcriptional activity of NF-κB. Interestingly, NF-κB activation in endothelial cells also mandatorily relies on the reductive arm of the LDH-1 reaction, i.e., NAD^+^ reduction in NADH + H^+^ (Vegran et al., 2011). In this complementary pathway, NADH fuels NAD(P)H oxidases (Noxs) that produce reactive oxygen species (ROS) to further inactivate IκBα. Thus, activation of both pyruvate-PHD and NADH-Nox signaling accounts for lactate-induced NF-κB activation in endothelial cells (Vegran et al., 2011). Lactate thereby supports autocrine interleukin-8 (IL-8) signaling and IL-8-induced tumor angiogenesis.

Active NF-κB is a dimer formed most often by the association of p50/NF-κB1 (that binds to DNA and contains a nuclear translocation domain) with p65/RelA (that contains transactivation domains). In tumor cells, NF-κB activation can result from mutations and aberrant kinase activities that essentially phosphorylate p65 on Ser536 (human sequence) to enhance p65 transcriptional activity (Viatour et al., 2005). Active NF-κB confers tumorigenic traits, including resistance to apoptosis and to therapy, stemness, and pro-angiogenic and metastatic capabilities (Chaturvedi et al., 2011). Because tumors accumulate high levels of lactate (Walenta and Mueller-Klieser, 2004) and lactate activates NF-κB in endothelial cells (Vegran et al., 2011), this study was aimed to test whether lactate could also activate NF-κB in tumor cells. We focused on oxidative tumor cells that do express the inward lactate transporter MCT1 and take up lactate, whereas glycolytic tumor cells are resistant to lactate signaling as they preferentially release lactate *via* MCT4 (passive transport) (De Saedeleer et al., 2012). We report that, precisely because of oxidative phosphorylation (OXPHOS), oxidative tumor cells do not activate NF-κB in response to lactate.

## Materials and methods

### Cells and reagents

HeLa human cervix adenocarcinoma cells (ATCC) were routinely cultured in RPMI 1640 containing Glutamax (Life Technologies catalogue #61870) supplemented with 10% FBS, 1% penicillin/streptomycin, 1 mM sodium pyruvate and 1X MEM non-essential amino acids (Life Technologies); SiHa human cervix squamous cell carcinoma cells and Cal27 human tongue squamous cell carcinoma cells (ATCC) in DMEM containing 4.5 g/L glucose and Glutamax (Life Technologies catalogue #61965) supplemented with 10% FBS and 1% penicillin/streptomycin; MCF7 human breast adenocarcinoma cells (ATCC) in MEM alpha supplemented with 10% FBS, 1% penicillin/streptomycin, 1 mM sodium pyruvate and 0.1% insulin; and human vein umbilical endothelial cells (HUVECs, Clonetics) in EBM2-MV medium (Cambrex). Where indicated, cells in fresh medium were treated with human TNFα (Miltenyi), sodium *L*-lactate (Sigma), *N*-acetyl-*L*-cysteine (NAC, Sigma), H_2_O_2_ (Sigma), rotenone (Sigma) or diethyl pyrocarbonate (DEPC, Sigma). To avoid modifications of extracellular pH, lactate was always used as a sodium salt in pH-buffered media. All experiments were performed in the presence of glucose.

### Metabolic assays

Oxygen consumption rates were determined on a Seahorse XF96 bioenergetic analyzer according to manufacturer's recommendations. Twenty thousand cells per well were plated 24-h before the experiment. NAD^+^/NADH ratios were determined using the NAD/NADH-Glo Assay from Promega according to manufacturer's instructions. All data were normalized for total protein content.

### Western blotting

After treatment, cells were washed once with ice-cold phosphate buffer saline (PBS) and lysed on ice in RIPA buffer containing protease and phosphatase inhibitors. Cell fractionation was performed using the Nuclear Extract Kit from Active Motif according to manufacturer's instructions. Cell lysates were frozen and thawed to ensure complete cell lysis and centrifuged at 13,000 g for 10 min, 4°C. Supernatants were collected and protein concentrations were determined using a Pierce BCA assay (Life Technologies). An equal amount of proteins were transferred in tubes containing Laemmli sample buffer (Sigma), heated for 5 min at 95°C, loaded in each well of homemade 1.5 mm-thick acrylamide gels, and separated by SDS-PAGE. Following electrophoresis, gels were blotted on PVDF membranes. After blocking for 1 h in TBST (10 mM Tris-HCl pH 8.0, 150 mM NaCl, 0.5% Tween-20) containing 5% bovine serum albumin (BSA), membranes were incubated with primary antibodies for 1 h, washed, and incubated for 1 h with secondary antibodies. Membranes were washed and incubated with ECL (Thermo Scientific) for chemiluminescent protein detection and quantification (ImageJ). Primary antibodies were rabbit polyclonals against Nox1 (Abcam #ab55831), Nox2 (Abcam #ab80508), Nox4 (Abcam #ab41886) and lamin A/C (Santa Cruz #sc-20681); rabbit monoclonals against total p65 (Cell Signaling #8242) and phospho-serine536-p65 (Cell Signaling #3033); and a mouse monoclonal against IκBα (Cell Signaling #4814). Secondary antibodies were a goat anti-rabbit (Jackson #111-035-003) and a goat anti-mouse (Jackson #115-035-003) coupled to horseradish peroxidase.

### NF-κB activity

NF-κB activity was measured using a luciferase reporter assay. Twenty-four hours before treatments, cells were transfected with pGL4.32 [luc2P/NF-κB-RE/Hygro], a Firefly luciferase-expressing plasmid driven by an artificial promoter containing five NF-κB consensus response elements and by pRL-Renilla-luciferase (Promega, constitutive expression for transfection normalization) using TransIT-2020 (Mirus Bio LLC). Reporter assays were performed with the dual luciferase kit (DLR) from Promega on a GloMax 96-well plate luminometer (Promega). All data were normalized for transfection efficiency by pRL Renilla luciferase.

### ROS measurement

Intracellular reactive oxygen species (ROS) were measured using 2′,7′-dichlorofluorescein diacetate (DCFH-DA, Sigma), as previously reported (Vegran et al., 2011). Signals were detected and quantified on a SpectraMax Imaging Cytometer i3. Data were normalized for total cell number.

### Statistics

All results are expressed as mean ± SEM. “*N*” refers to the number of independent experiments and “*n*” to the total number of replicates per treatment condition. Error bars are sometimes smaller than symbols. Student's *t*-test or One-way ANOVA (Tukey's or Bonferroni's *post-hoc* test) were used where convenient. *P* < 0.05 was considered to be statistically significant.

## Results

### Lactate does not activate NF-κB in oxidative tumor cells

**Figure 1:**
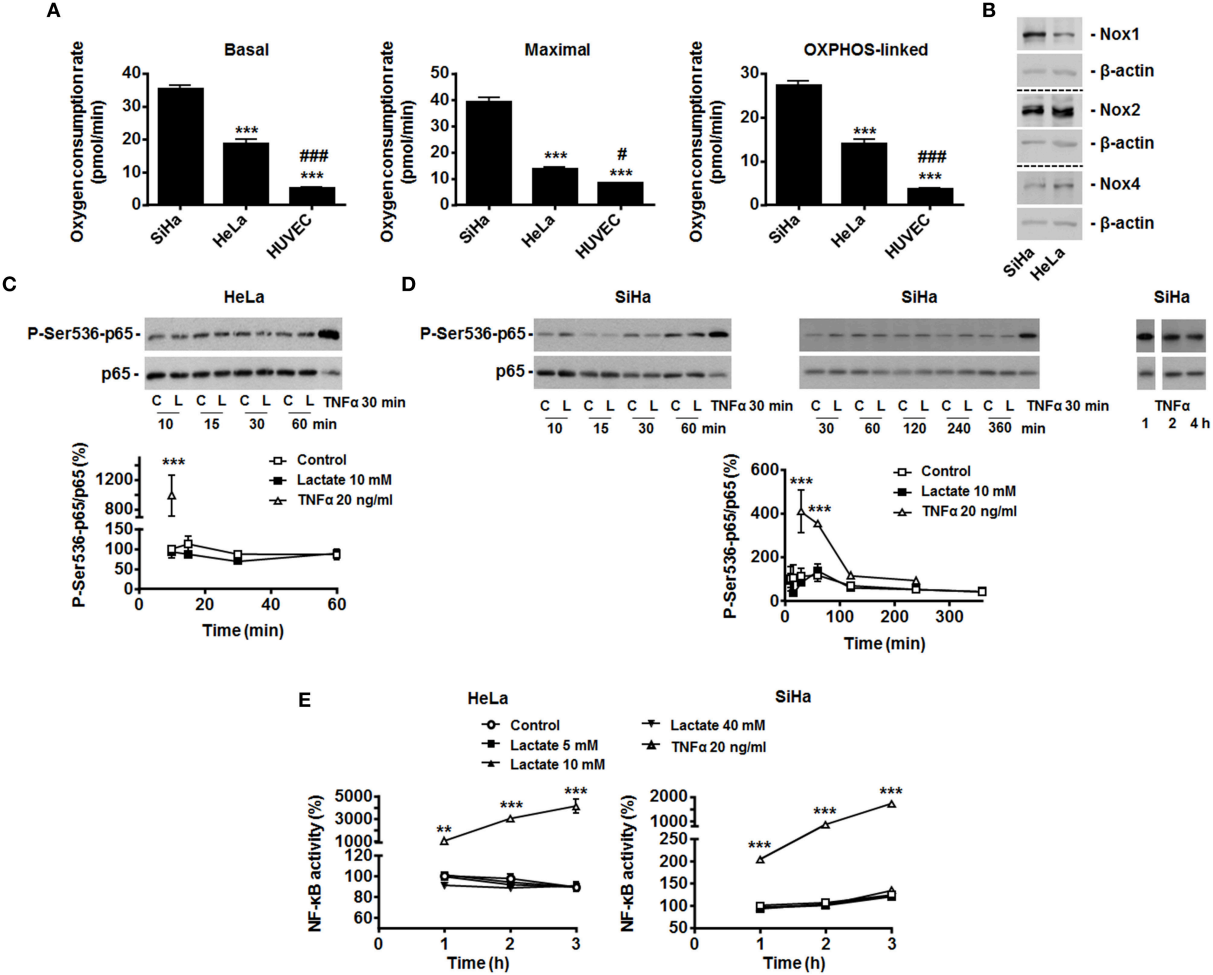
**Exogenous lactate does not activate NF-κB in oxidative HeLa and SiHa tumor cells**. **(A)** The basal, maximal and OXPHOS-dependent (i.e., rotenone and olygomycin-sensitive) oxygen consumption rates of SiHa, HeLa human tumor cells and human umbilical vein endothelial cells (HUVEC) were determined using Seahorse bioanalysis (*n*≥6). **(B)** Representative western blots showing Nox1, Nox2, Nox4 and β-actin expression in HeLa and SiHa cells. **(C)** HeLa cells were treated with vehicle (control, C), 10 mM of sodium *L*-lactate (lactate, L) or 20 ng/ml of TNFα (positive control) for the indicated periods of time. The phosphorylation of p65 on serine 536 (p-Ser536-p65) and total p65 expression were detected using western blotting in the lysates of the cells. Treatment with TNFα (20 ng/ml) served as positive control. A representative blot is shown, and the graph depicts p-Ser536-p65/total p65 used as a marker of NF-κB activity (*N* = 3; *n* = 9) (Viatour et al., 2005). **(D)** As in **(C)** but using SiHa cells (*N* = 9; *n* = 9). **(E)** HeLa and SiHa cells were treated with vehicle (control), increasing doses of lactate or TNFα. NF-κB activity was determined using a dual luciferase reporter assay (*n* = 4 all). All data represent means ± SEM. ^**^P < 0.01, ^***^P < 0.005 vs. SiHa **(A)** or control **(C–E)**; ^#^*P* < 0.05, ^*###*^*P* < 0.005 vs. HeLa **(A)**.

We selected SiHa and HeLa human cervix cancer cells as models: both cell types have been well described to primarily rely on an oxidative metabolism, to express MCT1 and to be sensitive to lactate-induced PHD inhibition (inward lactate gradient) (Sonveaux et al., 2008; De Saedeleer et al., 2012). Compared to WiDr human colon cancer cells that are highly glycolytic and resistant to lactate signaling (outward lactate gradient) (De Saedeleer et al., 2012), we first found that SiHa and HeLa are more oxidative than the human vein umbilical endothelial cells (HUVECs) that initially served to identify the lactate to NF-κB pathway (Vegran et al., 2011) (Figure 1A). HUVECs express Nox2 and Nox4 (Van Buul et al., 2005), and SiHa and HeLa cells did express Nox1, Nox2, and Nox4 (Figure 1B).

To test the effects of exogenous lactate on NF-κB activity, we first analyzed p65 Ser536-phosphorylation/activation in HeLa cells exposed to exogenous lactate. Lactate was used as a sodium salt (to avoid lactate-independent pH responses) at a 10 mM concentration (to match with the average lactate concentration found in human tumors) (Walenta and Mueller-Klieser, 2004), i.e., conditions upon which lactate inhibits PHDs in these cells (De Saedeleer et al., 2012). Time-course measurements over 1-h showed no increase in the phospho-Ser536-p65/total p65 ratio (Figure 1C), whereas TNFα used as a positive control did trigger p65 Ser536-phosphorylation. Total p65 levels were unaffected by lactate but decreased with TNFα treatment (Figure 1C). In SiHa cells, similarly to what was observed in HeLa, time-course measurements up to 6 h showed a stable phospho-Ser536-p65/total p65 ratio (Figure 1D). TNFα increased p65 phosphorylation in these cells.

That exogenous lactate is unable to trigger NF-κB activity in oxidative tumor cells was verified using a specific dual luciferase reporter assay. Increasing doses of lactate were used, ranging from 0 to 40 mM, the latter corresponding to the highest dose of lactate ever detected in human tumors (Walenta and Mueller-Klieser, 2004). Up to 3 h after treatment, no significant changes of NF-κB activity were detected in HeLa and SiHa cells (Figure 1E). Still, TNFα induced a 10 to 40-fold increase in NF-κB activity in these assays.

### Oxidative tumor cells are capable of activating NF-κB

**Figure 2:**
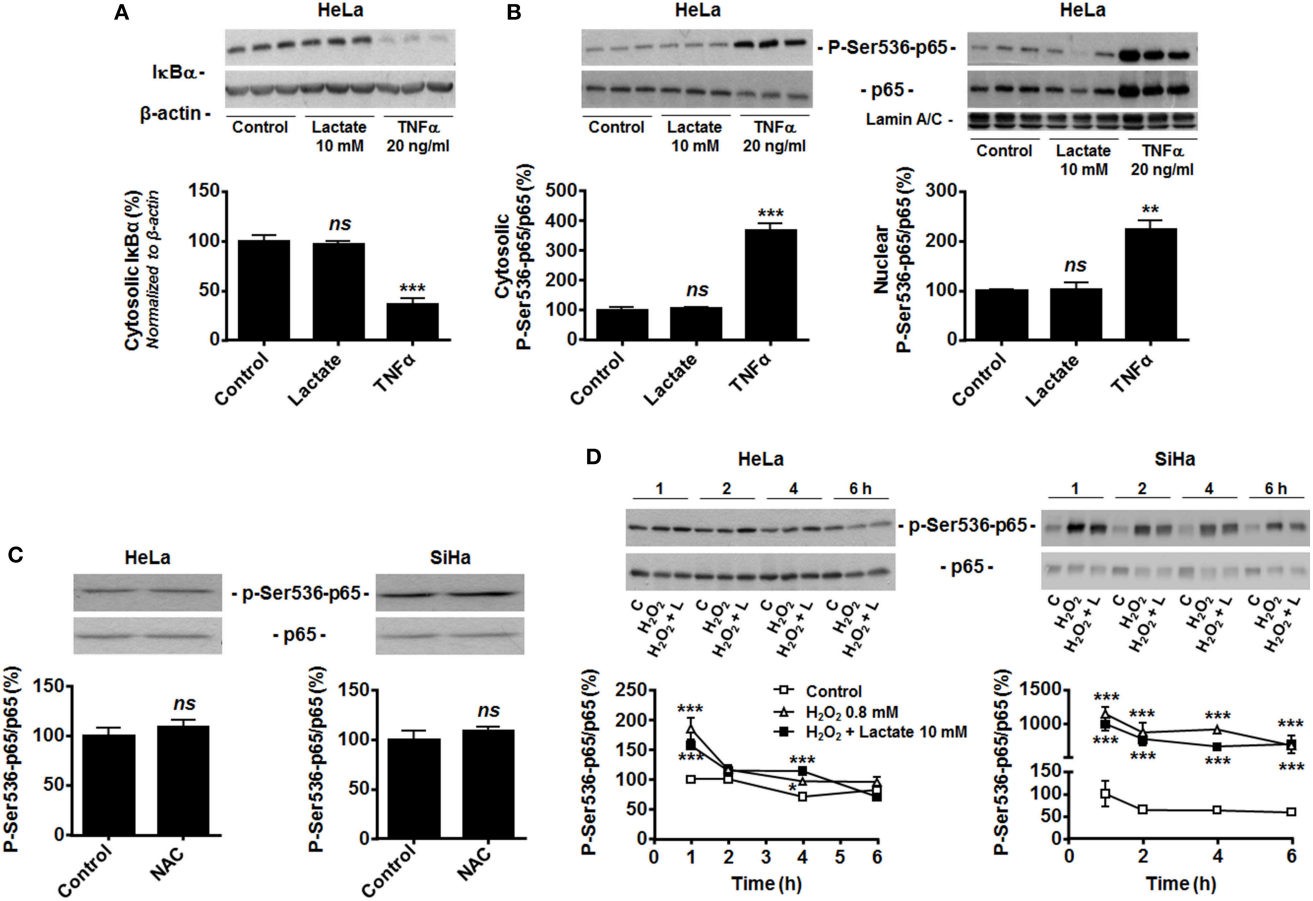
**Hydrogen peroxide activates NF-κB in oxidative tumor cells**. **(A)** Inhibitor of NF-κB α (IκBα) was detected in the lysates of HeLa cells treated during 30 min with vehicle (control), lactate (10 mM) or TNFα (20 ng/ml). A representative western blot is shown and the graph depicts IκBα expression (*N* = 3; *n* = 9). **(B)** The phosphorylation of p65 on serine 536 (p-Ser536-p65) and total p65 expression were detected using western blotting in the cytosolic (*left panel*, *N* = 3; *n* = 9) and lamin A/C-positive nuclear (*right panel*, *N* = 3; *n* = 9); lysates of HeLa cells treated as in **(A)**. Representative blot is shown, and the graph depicts p-Ser536-p65/total p65 ratios. **(C)** HeLa (*left panel*, *N* = 3; *n* = 9) and SiHa (*right panel*, *N* = 3; *n* = 9) cells were treated for 20 min with vehicle (control) or 10 mM of *N*-acetyl-*L*-cysteine (NAC), after which p-Ser536-p65 and total p65 were detected in cell lysates. Representative blots are shown, and graphs depict p-Ser536-p65/total p65 ratios. **(D)** HeLa (*left panel*, *N* = 3; *n* = 9) and SiHa (*right panel*, *N* = 3; *n* = 9) cells were treated during increasing amounts of time with vehicle (control, C), H_2_O_2_ (0.8 mM) or H_2_O_2_ (0.8 mM) and lactate (10 mM). p-Ser536-p65 and total p65 were detected in cell lysates. Representative western blots are shown, and graphs depict p-Ser536-p65/total p65 ratios. All data represent means ± SEM. ^*^P < 0.05, ^**^P < 0.01, ^***^P < 0.005, *ns P* > 0.05 vs. control.

In endothelial cells, lactate-induced NF-κB activation depends on two signaling pathways that must be activated together, with pyruvate and NADH as key intermediates (Vegran et al., 2011). Previous data from our laboratory clearly evidenced that lactate is efficiently converted to pyruvate in both HeLa and SiHa cells (Sonveaux et al., 2008; De Saedeleer et al., 2012, 2014) and that pyruvate then inhibits PHDs in these cells (De Saedeleer et al., 2012). Despite this, HeLa cells did not reduce IκBα expression (Figure 2A) and did not translocate p65 to the nucleus (Figure 2B) in response to lactate, whereas they did so in response to TNFα.

Many tumor cells display constitutive NF-κB activity (Chaturvedi et al., 2011), which could make them insensitive to further induction by lactate. However, HeLa and SiHa cells were highly sensitive to TNFα (Figures 1C–E, 2A,B). Because both cell lines do express Nox isoforms (Figure 1B), we tested whether high basal oxidative stress could account for constitutive NF-κB activation. It was not the case as *N*-acetyl-*L*-cysteine (NAC, 10 mM) did not decrease the phospho-Ser536-p65/total p65 ratio (Figure 2C). Conversely, exposure of the cells to 0.8 mM H_2_O_2_ did increase p65 phosphorylation in both cell lines (Figure 2D). In HeLa cells, H_2_O_2_ effects were maximal at 1-h, followed by a rapid decline down to basal levels. In SiHa cells, significant induction was detected from 1 to 6-h. In both cell lines, lactate had no additive effects over H_2_O_2_ (Figure 2D). These data collectively suggest that oxidative tumor cells are insensitive to lactate-induced NF-κB activation because of a failure to trigger NADH-Nox-ROS signaling.

### Because OXPHOS consumes cytosolic NADH, oxidative hela tumor cells do not activate NF-κB in response to lactate

**Figure 3:**
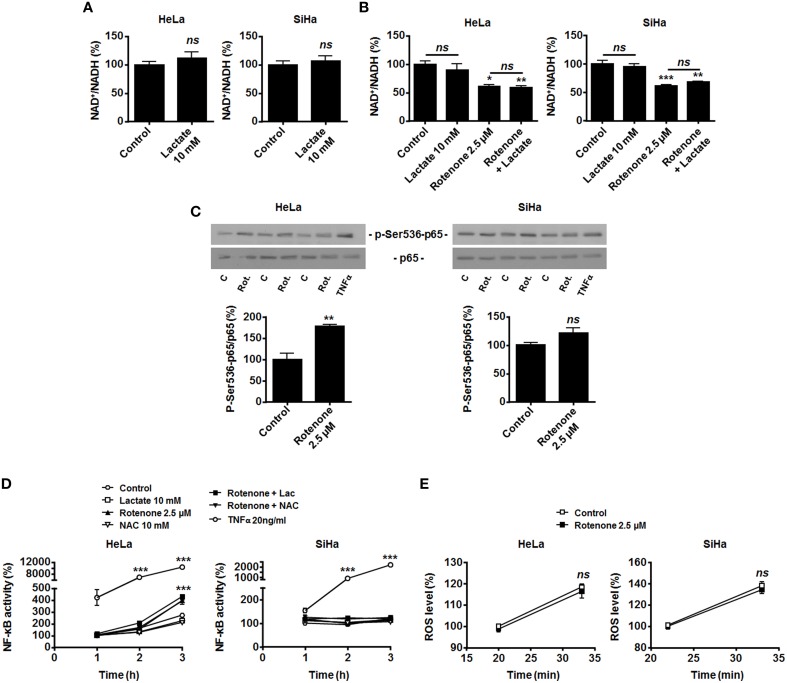
**OXPHOS inhibition by rotenone activates NF-κB in oxidative HeLa tumor cells**. **(A)** HeLa (*left panel*, *N* = 3; *n* = 9) and SiHa (*right panel*, *N* = 3; *n* = 9) cells were treated for 30 min with 10 mM of lactate, and NAD^+^/NADH ratios were determined. **(B–D)** HeLa (*left panels*) and SiHa (*right panels*) cells were treated for 30 min with vehicle (control, C), 10 mM of lactate (L), 2.5 μM of rotenone (Rot.), 10 mM of *N*-acetyl-*L*-cysteine (NAC), 20 ng/ml of TNFα or combinations thereof. **(B)** NAD^+^/NADH ratios were determined (*N* = 3; *n* = 9 all). **(C)** p-Ser536-p65 and total p65 expression were detected using western blotting. Representative blots are shown, and graphs depict p-Ser536-p65/total p65 ratios (*N* = 3; *n* = 9 all). **(D)** NF-κB activity was determined using a dual luciferase reporter assay (*N* = 3; *n* = 12 all). **(E)** HeLa (*left panel*, *N* = 3; *n* = 12) and SiHa (*right panel N* = 3; *n* = 12) cells were treated for the indicated time periods with vehicle (control) or rotenone (2.5 μM), after which intracellular ROS levels were measured using the fluorescent probe DCFH-DA. All data represent means ± SEM. ^*^P < 0.05, ^**^P < 0.01, ^***^P < 0.005, *ns P* > 0.05 vs. control.

HeLa and SiHa cells efficiently convert lactate to pyruvate, which implies the effective conversion of NAD^+^ to NADH + H^+^ by LDH-1 (Sonveaux et al., 2008; De Saedeleer et al., 2012, 2014). However, it does not obligatorily mean that NADH is made available for Noxs. In agreement with an alternative use of NADH, we found that treating HeLa and SiHa cells with 10 mM of lactate did not decrease the NAD^+^/NADH ratio (Figure 3A). This observation indicated that oxidative tumor cells use NADH for another purpose than fueling Noxs.

Compared to endothelial cells that generate most of their energy through glycolysis (Figure 1A and De Bock et al., 2013), we reasoned that oxidative tumor cells would have high OXPHOS avidity for NADH. NADH of cytosolic origin can indeed be imported into mitochondria *via* the malate-aspartate shuttle to fuel the electron transport chain (ETC), providing additional proton motor force for ATP production (Ying, 2008). To test whether LDH-1-derived NADH is preferentially used for mitochondrial supply, we first treated the cells with rotenone to fully inhibit ETC at complex I, where NADH is used. In the absence of lactate, OXPHOS impairment significantly decreased the NAD^+^/NADH ratio (Figure 3B), induced Ser536-p65 phosphorylation (Figure 3C) and activated NF-κB in HeLa cells. Rotenone also significantly decreased the NAD^+^/NADH ratio in SiHa cells (Figure 3B), which was however not associated to NF-κB induction (Figures 3C,D). Interestingly, in HeLa and SiHa cells, TNFα triggered NF-κB much more efficiently than rotenone (Figures 3C,D). While ROS are downstream effectors of TNFα (Chu, 2013) and ROS activate NF-κB (Gloire et al., 2006), full ETC inhibition by rotenone did not modify ROS levels in the cells (Figure 3E) and NF-κB activation by rotenone was ROS-independent as it could not be blocked by NAC (Figure 3D). Supplying exogenous lactate to OXPHOS-impaired cells did not further activate NF-κB (Figure 3D).

**Figure 4:**
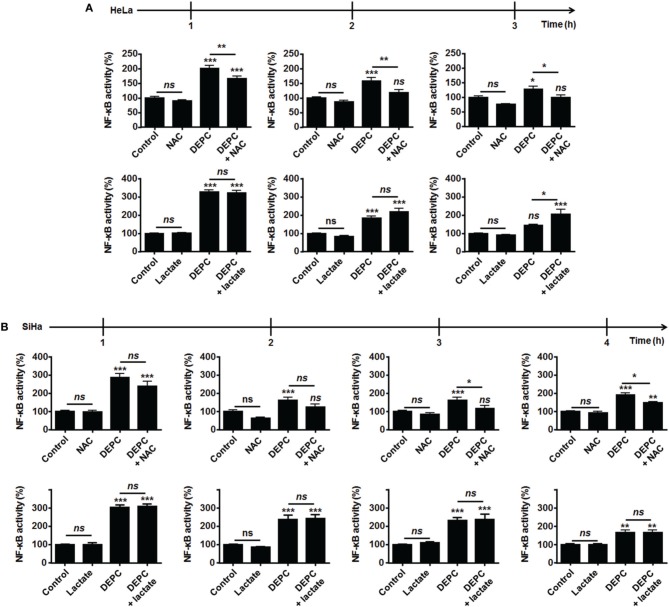
**Inhibition of the malate-aspartate shuttle restores the ability of lactate to activate NF-κB in oxidative HeLa tumor cells**. **(A,B)** NF-κB activity was determined using a dual luciferase reporter assay. HeLa (**A**, *N* = 3; *n* = 12), SiHa (**B**, *N* = 3; *n* = 12) cells were treated for the indicated amounts of time with vehicle (control), 10 mM of lactate, 1 mM of diethyl pyrocarbonate (DEPC, a pharmacological inhibitor of the mitochondrial glutamate-aspartate antiporter) (Samartsev et al., 1997), 10 mM of *N*-acetyl-*L*-cysteine (NAC) or combinations thereof. All data represent means ± SEM. ^*^P < 0.05, ^**^P < 0.01, ^***^P < 0.005, *ns P* > 0.05 vs. control or as indicated.

**Figure 5:**
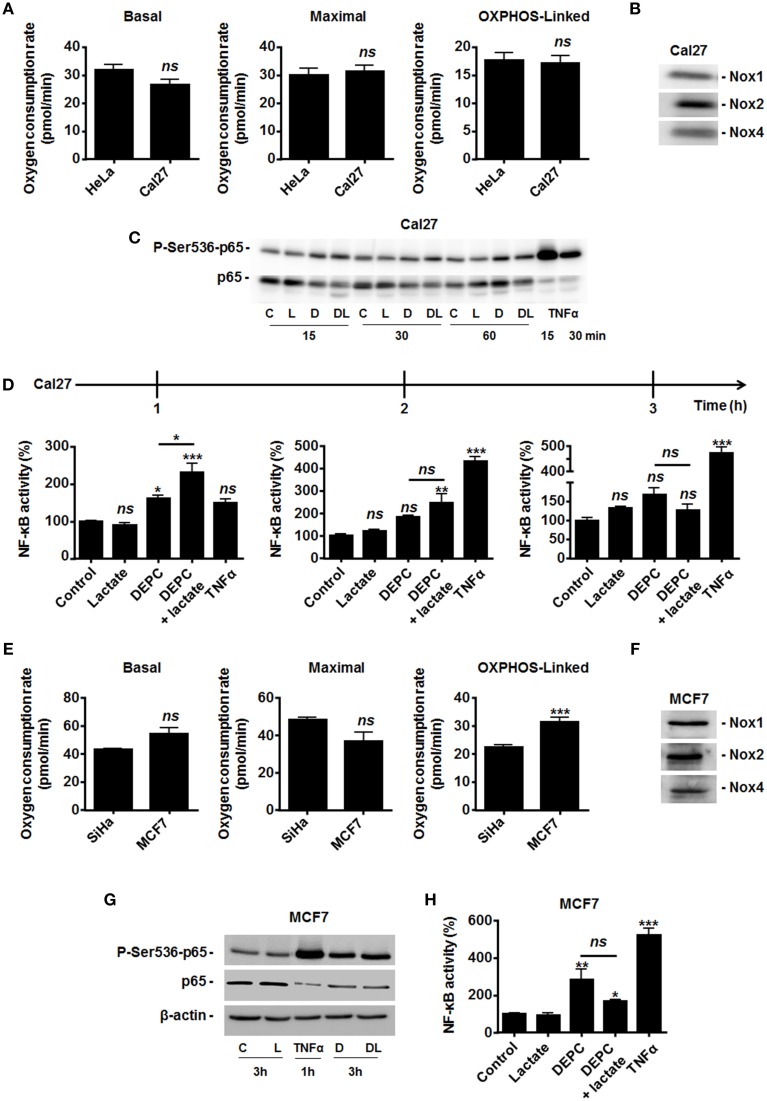
**Inhibition of the malate-aspartate shuttle restores the ability of lactate to activate NF-κB in oxidative Cal27 tumor cells**. **(A)** The basal, maximal and OXPHOS-dependent oxygen consumption rates of HeLa and Cal27 human tumor cells were determined using Seahorse bioanalysis (*n*≥6). **(B)** Representative western blots showing Nox1, Nox2, Nox4 expression in Cal27 cells. **(C)** Cal27 cells were treated for the indicated amounts of time with vehicle (control, C), 1 mM of diethyl pyrocarbonate (DEPC, D), 10 mM Lactate (L), the combination of DEPC and lactate (DL), or TNFα (20 ng/ml), after which p-Ser536-p65 and total p65 were detected in cell lysates. Representative western blots are shown. **(D)** Cal27 cells were treated as in **(C)** except that NF-κB activity was determined using a dual luciferase reporter assay 1, 2, and 3 h after treatment (*n* = 4). **(E)** The basal, maximal and OXPHOS-dependent oxygen consumption rate of SiHa and MCF7 human tumor cells were determined using Seahorse bioanalysis (*n*≥6). **(F)** Representative western blots showing Nox1, Nox2, Nox4 expression in MCF7 cells. **(G)** MCF7 cells were treated for 3 h with vehicle (control, C), 1 mM of DEPC (D), 10 mM of L-Lactate (L) or combination (DL), after which p-Ser536-p65, total p65 and β-actin were detected in cell lysates. A 1-h treatment with TNFα was used as positive control. Representative western blots are shown. **(H)** Same as in **(G)** except that NF-κB activity was determined using a dual luciferase reporter assay (*n* = 4). All data represent means ± SEM. ^*^P < 0.05, ^**^P < 0.01, ^***^P < 0.005, *ns P* > 0.05 vs. control or as indicated.

Because MCT1-mediated lactate uptake is a passive process depending on the gradient of lactate across the plasma membrane and lactate must be converted to pyruvate to fuel the TCA cycle and for intracellular signaling (Dhup et al., 2012), inhibiting OXPHOS decreases lactate uptake by oxidative tumor cells (Sonveaux et al., 2008). Therefore, we finally reasoned that inhibiting the mitochondrial use of NADH could be sufficient to restore the capacity of lactate to activate NF-κB. To this aim, we used diethyl pyrocarbonate (DEPC) (Samartsev et al., 1997), an inhibitor of the mitochondrial glutamate-aspartate antiporter that composes the malate-aspartate shuttle indispensable for the mitochondrial import of cytosolic NADH (Dionisi et al., 1974; Greenhouse and Lehninger, 1977). In HeLa (Figure 4A) and in SiHa cells (Figure 4B), DEPC (1 mM) activated NF-κB, which was repressed using NAC. It clearly indicated that the mitochondrial use of NADH prevents ROS-dependent NF-κB activation in these cells. Time-course experiments further showed that exogenous lactate activated NF-κB in HeLa cells upon inhibition of the malate-aspartate shuttle, as detected 3-h after treatment (Figure 4A). Comparatively, we detected no additional effect of lactate on DEPC-induced NF-κB activation in SiHa cells (Figure 4B). These data were confirmed using two additional tumor cell lines: Cal27 human tongue squamous cell carcinoma cells and MCF7 human breast adenocarcinoma cells. Cal27 cells were equally oxidative when compared to HeLa (Figure 5A). They did express Nox1, Nox2, and Nox4 (Figure 5B) but did not phosphorylate p65 on Ser536 (Figure 5C) and did not activate NF-κB (Figure 5D) in the presence of exogenous lactate. Inhibition of the mitochondrial glutamate-aspartate antiporter by DEPC increased P-Ser536-p65 expression (Figure 5C) and NF-κB activity, with a further activation of NF-κB by lactate detected 1 and 2 h after DEPC treatment (Figure 5D). Comparatively, MCF7 cells were equally oxidative when compared to SiHa (Figure 5E). As for all the oxidative tumor cells tested in this study, they did express Nox1, Nox2, and Nox4 (Figure 5F) but did not activate the NF-κB pathway in response to lactate (Figures 5G,H). DEPC increased P-Ser536-p65 expression (Figure 5G) and NF-κB activity (Figure 5H) with no additional effect of lactate 3 h after DEPC treatment, thus recapitulating the behavior of SiHa cells.

## Discussion

Oncometabolite lactate is a tumor growth-promoting factor that can be used as a metabolic fuel and as a signaling agent by various cell types. Based on recent findings in endothelial cells (Vegran et al., 2011, 2012), this study explored the possibility that lactate would activate NF-κB in oxidative tumor cells. We report that, despite the fact that it is oxidized to pyruvate (together with the reduction of NAD^+^ to NADH + H^+^) (Lu et al., 2002, 2005; Sonveaux et al., 2008; De Saedeleer et al., 2012), lactate does not activate NF-κB in oxidative tumor cells.

With respect to lactate signaling in cancer, Lu et al. (2002, 2005) initially showed that lactate is a hypoxia mimetic capable of activating transcription factor hypoxia-inducible factor-1 (HIF-1) in normoxic tumor cells. These authors reported that lactate oxidation to pyruvate by LDH-1 supports a competition between pyruvate and αKG that inhibits HIF PHDs. Comparatively, because lactate uptake is a passive process (facilitated by MCTs and driven by the gradient of lactate across the plasma membrane), glycolytic tumor cells that produce lactate from pyruvate do not activate HIF-1 in response to exogenous lactate (De Saedeleer et al., 2012). PHDs are iron-containing enzymes that use oxygen and αKG to catalyze the hydroxylation of 2 proline residues in the oxygen-dependent domain (ODD) of HIF-1 subunit α. While under normoxia ODD proline hydroxylation normally targets HIF-1α for polyubiquitylation and proteasome-mediated degradation, Lu et al. elegantly showed that cell exposure to exogenous lactate interferes with this pathway and accounts for normoxic HIF-1 activation. Consequently, lactate induces the expression of several HIF-1 target genes, among which vascular endothelial growth factor A (VEGFA) in normoxic oxidative tumor cells (Lu et al., 2005; De Saedeleer et al., 2012) and VEGF receptor-2 (VEGFR2) in normoxic endothelial cells (Sonveaux et al., 2012) and macrophages (Colegio et al., 2014) are key contributors to lactate-induced angiogenesis. In response to lactate, endothelial cells further increase their production of basic fibroblast growth factor (bFGF) through a mechanism that indirectly depends on HIF-1 (Sonveaux et al., 2012), and tumor-associated macrophages are polarized to the M2 protumoral phenotype (Colegio et al., 2014). Together, HIF-1 activation by lactate was found to trigger angiogenesis *in vivo* (De Saedeleer et al., 2012; Sonveaux et al., 2012), which for anticancer effects can be blocked by MCT1 inhibitors and, conversely, can be exploited therapeutically in wounds to accelerate the healing process (Porporato et al., 2012; Chereddy et al., 2014, 2015).

**Figure 6:**
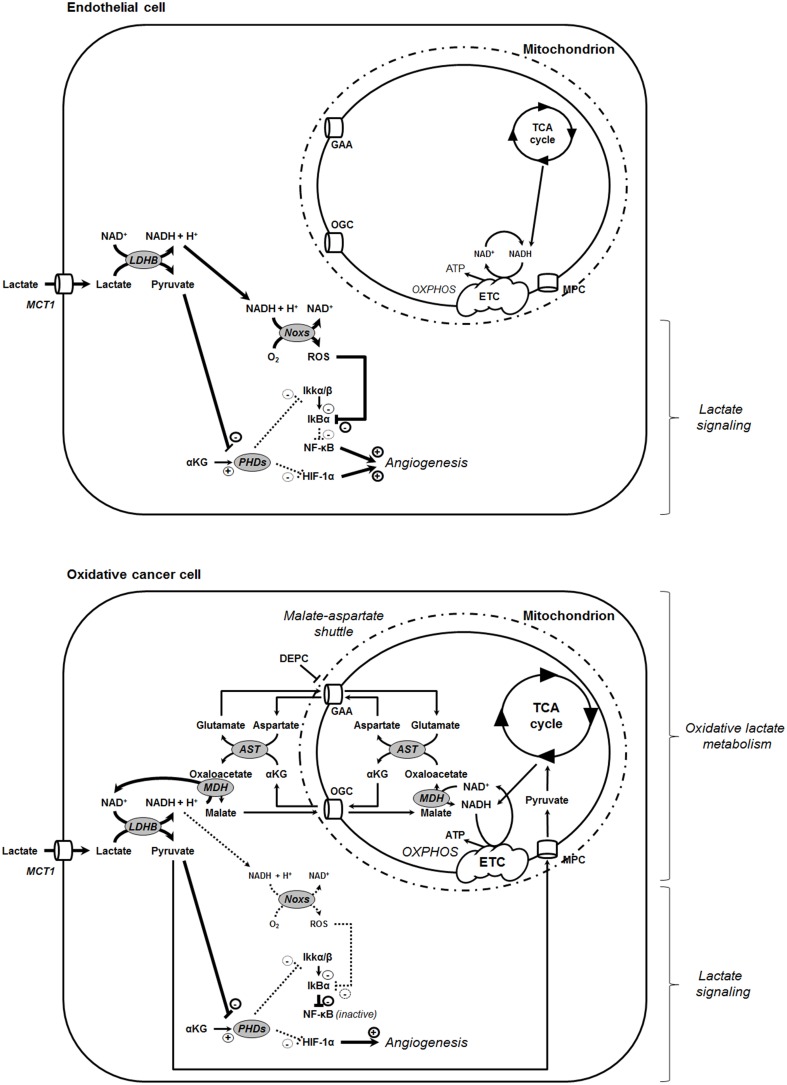
**Model showing that the oxidative use of NADH opposes NF-κB activation by lactate in oxidative tumor cells**. In tumors, endothelial cells (**top**) and oxidative tumor cells (**bottom**) take up lactate, a process facilitated by the passive lactate-proton symporter monocarboxylate transporter 1 (MCT1). Both cell types convert lactate + NAD^+^ to pyruvate + NADH + H^+^ intracellularly (the lactate dehydrogenase B [LDHB] reaction). Pyruvate competes with α-ketoglutarate (αKG) to inhibit prolylhydroxylases (PHDs), resulting e.g., in the stabilization of hypoxia-inducible factor-1 subunit α (HIF-1α) (Vegran et al., 2011; De Saedeleer et al., 2012; Sonveaux et al., 2012). Endothelial cells do not use lactate-derived pyruvate and NADH as oxidative fuels (Sonveaux et al., 2012), thus further rendering NADH available to fuel NAD(P)H oxidases (Noxs) (Vegran et al., 2011). Together with pyruvate-mediated PHD inhibition, the production of reactive oxygen species (ROS) by Nox accounts for lactate-induced activation of nuclear factor-κB (NF-κB) in these cells. Comparatively, oxidative tumor cells use lactate (Sonveaux et al., 2008) and NADH (this study) as oxidative fuels. It renders these cells insensitive to lactate-induced NF-κB activation. NADH influx in mitochondria is controlled by the malate-aspartate shuttle gated by the oxoglutarate carrier (OGC, a malate-αKG exchanger) and by the glutamate-aspartate antiporter (GAA). Consequently, inhibiting the malate-aspartate shuttle can restore the ability of lactate to activate NF-κB in oxidative tumor cells, as illustrated in this study with HeLa cells treated by GAA inhibitor diethyl pyrocarbonate (DEPC). Other abbreviations: AST, aspartate aminotransferase; ETC, electron transport chain; IκBα, inhibitor and Cal27 of NF-κB α; Ikkα/β, inhibitor of NF-κB kinase α/β; MDH, malate dehydrogenase; MPC, mitochondrial pyruvate carrier; OXPHOS, oxidative phosphorylation.

NF-κB also contributes to lactate-induced angiogenesis in tumors. Indeed, when in addition to pyruvate inhibiting PHDs, NADH aliments ROS production by Noxs, IκBα is ultimately degraded, releasing p65 from its inhibitory interaction with IκBα (Vegran et al., 2011). Thus, both pyruvate-mediated inhibition of PHDs and NADH-mediated ROS generation by Noxs are simultaneously and mandatorily required for lactate to activate NF-κB in endothelial cells (Figure 6). Generally speaking, endothelial cells have a glycolytic rate that can be as high as glycolytic tumor cells (De Bock et al., 2013), which is in frame with their function of delineating oxygen-delivering blood vessels. Accordingly, we illustrate here that the HUVECs that were instrumental to identify lactate-induced NF-κB activation (Vegran et al., 2011) are much more glycolytic than SiHa and HeLa tumor cells that served as oxidative archetypes to identify lactate-induced HIF-1 activation (De Saedeleer et al., 2012). Endothelial cells can use lactate to activate HIF-1 (Sonveaux et al., 2012) and NF-κB (Vegran et al., 2011), but not as a major fuel for cell survival in the absence of glucose (Sonveaux et al., 2012). Comparatively, oxidative tumor cells can use lactate as a major fuel (Sonveaux et al., 2008) and to activate HIF-1 (De Saedeleer et al., 2012), but not to activate NF-κB (this study). Highly glycolytic tumor cells do not use exogenous lactate as a fuel or as a signaling agent because their abundant release of lactate opposes lactate uptake (MCTs are passive transporters) (De Saedeleer et al., 2012). At least in oxidative HeLa and Cal27 cells, the lack of response of the NF-κB pathway to lactate can be explained by a preferential use of NADH by OXPHOS in mitochondria rather than to aliment Noxs: inhibition of the mitochondrial uptake of NADH by DEPC restored NF-κB activation by lactate in these cells (Figures 4, 5). While DEPC *per se* already activated NF-κB in HeLa and Cal27 cells, lactate exerted an additional effect, collectively indicating that it is indeed the cytosolic availability of NADH that drives NF-κB activity in these models. Using HeLa, we further evidenced that the activity of DEPC was ROS-mediated as it could be blocked by NAC. Similarly, DEPC activated NF-κB in SiHa and MCF7 cells. However, lactate did not further activate the pathway, which we attribute to a better efficacy and longer time of action of DEPC in these cells that are twice more oxidative than HeLa and Cal27. Importantly, tumors including those established *in vivo* with oxidative tumor cells are heterogeneous metabolically (Sonveaux et al., 2008; Payen et al., 2015). Compared to the archetypic oxidative cell lines used in this study, microenvironmental influences on tumor cell metabolism could thus generate a range of intermediate metabolic phenotypes with different responsiveness of the NF-κB pathway to lactate. It is also important to stress that TNFα can be secreted locally in tumors essentially by macrophages, resulting in a receptor-mediated activation of NF-κB that promotes tumor growth during cancer-associated chronic inflammation (for details, see reference Muntané, 2011).

Full ETC blockade by rotenone activated NF-κB independently of ROS in HeLa cells, with no additional effect of lactate. It failed to do so in SiHa cells. The lack of effect of lactate can be explained by the fact that lactate uptake is a passive process driven by the gradient of lactate across the plasma membrane (Dhup et al., 2012; Halestrap and Wilson, 2012): following the mass action law and as demonstrated experimentally (Sonveaux et al., 2008; De Saedeleer et al., 2012), full ETC inhibition can inhibit lactate uptake and activity because products of the LDH1 reaction are not consumed any more. However, at this stage, we have no consistent explanation for the effects of rotenone alone. A path to explore could be the capability of rotenone to trigger TNFα release and signaling, as recently observed in microglial cells (Yuan et al., 2013).

Finally, we believe that the major finding of this study is that, in oxidative tumor cells, both pyruvate (Sonveaux et al., 2008) and NADH produced following lactate oxidation by LDH1 primarily serve to fuel mitochondrial metabolism (Figure 6). This observation offers a striking similarity with the glycolytic pathway that, when coupled to mitochondrial metabolism, fuels the TCA cycle with pyruvate produced by pyruvate kinase, and OXPHOS with NADH produced by glyceraldehyde-3-phosphate dehydrogenase (GAPDH). This finding positions the malate-aspartate shuttle as a key contributor to the oxidative metabolism of cancer cells and LDH1 as a potential target to simultaneously inhibit lactate metabolism and signaling.

### Conflict of interest statement

The authors declare that the research was conducted in the absence of any commercial or financial relationships that could be construed as a potential conflict of interest.
